# Epigenetically altered miR-193b targets cyclin D1 in prostate cancer

**DOI:** 10.1002/cam4.486

**Published:** 2015-07-01

**Authors:** Kirsi M Kaukoniemi, Hanna E Rauhala, Mauro Scaravilli, Leena Latonen, Matti Annala, Robert L Vessella, Matti Nykter, Teuvo L J Tammela, Tapio Visakorpi

**Affiliations:** 1 Institute of Biosciences and Medical Technology - BioMediTech, University of TampereTampere, Finland; 2 Fimlab Laboratories, Tampere University HospitalTampere, Finland; 3 Department of Urology, University of WashingtonSeattle, Washington; 4 Department of Urology, University of Tampere and Tampere University HospitalTampere, Finland

**Keywords:** Cyclin D1, micro-RNA, prostate cancer

## Abstract

Micro-RNAs (miRNA) are important regulators of gene expression and often differentially expressed in cancer and other diseases. We have previously shown that miR-193b is hypermethylated in prostate cancer (PC) and suppresses cell growth. It has been suggested that miR-193b targets cyclin D1 in several malignancies. Here, our aim was to determine if miR-193b targets cyclin D1 in prostate cancer. Our data show that miR-193b is commonly methylated in PC samples compared to benign prostate hyperplasia. We found reduced miR-193b expression (*P *<* *0.05) in stage pT3 tumors compared to pT2 tumors in a cohort of prostatectomy specimens. In 22Rv1 PC cells with low endogenous miR-193b expression, the overexpression of miR-193b reduced *CCND1*mRNA levels and cyclin D1 protein levels. In addition, the exogenous expression of miR-193b decreased the phosphorylation level of RB, a target of the cyclin D1-CDK4/6 pathway. Moreover, according to a reporter assay, miR-193b targeted the 3’UTR of *CCND1* in PC cells and the *CCND1* activity was rescued by expressing *CCND1* lacking its 3’UTR. Immunohistochemical analysis of cyclin D1 showed that castration-resistant prostate cancers have significantly (*P *=* *0.0237) higher expression of cyclin D1 compared to hormone-naïve cases. Furthermore, the PC cell lines 22Rv1 and VCaP, which express low levels of miR-193b and high levels of *CCND1*, showed significant growth retardation when treated with a CDK4/6 inhibitor. In contrast, the inhibitor had no effect on the growth of PC-3 and DU145 cells with high miR-193b and low *CCND1* expression. Taken together, our data demonstrate that miR-193b targets cyclin D1 in prostate cancer.

## Introduction

Prostate cancer (PC) is the second most frequently diagnosed cancer type and the sixth leading cause of cancer-related deaths in males worldwide 1. Localized PC can be cured with prostatectomy and/or radiation therapy. However, there is no curative treatment for advanced and castration-resistant disease 2. The use of prostate-specific antigen (PSA) for the screening of asymptomatic men for prostate cancer is known to reduce the disease-specific mortality, but screening is associated with overdiagnosis 3. Therefore, there is a strong interest to find additional markers and therapeutic targets for the diagnosis and treatment of prostate cancer. MicroRNAs (miRNAs) could serve as such markers or as drug targets 4.

miRNAs are small, ∼22 nucleotide long noncoding RNA molecules that were first discovered in *Caenorhabditis elegans* 5. Since their discovery, miRNAs have been found to be important regulators of gene expression in other organisms as well 5,6. miRNAs modulate gene expression by binding to a complementary sequence in the 3’ untranslated region (3’UTR) of target messenger RNAs (mRNAs). The binding of a miRNA leads to the degradation of the mRNA molecule or alternatively, to the suppression of mRNA translation, depending on the level of complementary binding 7. Because one miRNA can have multiple targets and one mRNA molecule can be targeted by many miRNAs, miRNAs form a complex level of regulation of gene expression. In addition to genetic changes (chromosomal rearrangements, deletions, amplifications, and mutations), epigenetic events such as aberrant promoter hypermethylation, global hypomethylation, and posttranscriptional histone modification may also cause the dysregulation of miRNAs in cancer 7. Several miRNAs are deregulated in prostate cancer and have been shown to affect apoptosis, cell cycle, intracellular signaling, DNA repair, adhesion/migration, and androgen signaling (reviewed in 4).

We previously showed that hsa-miR-193b-3p (aka miR-193b) may function as an epigenetically regulated tumor suppressor in prostate cancer 8. Using bisulfite sequencing we showed that miR-193b is hypermethylated in some PC cell lines, with the 22Rv1 cell line being the most heavily methylated resulting in the loss of miR-193b expression in the cells. Transient transfection of pre-miR-193b into 22Rv1 cells caused significant growth reduction due to a decreased fraction of cells in S-phase of the cell cycle.

There are several suggested target genes for miR-193b in different cancers, for example, *CCND1, ETS1*, and *KIT* 9–16. The cyclin D1-encoding gene, *CCND1*, is targeted by miR-193b in hepatocellular carcinoma 10, melanoma 11, and pancreatic cancer 14. Cyclin D1 is frequently aberrantly expressed in cancer. It regulates the expression of genes that are involved in DNA replication and the DNA damage checkpoint. The best known function of cyclin D1 is most likely its catalytic function as a regulatory partner for the cyclin-dependent kinases (CDKs) 4 and 6. In response to cellular stimuli, cyclin D1 activates CDKs 4 and 6, which in turn phosphorylate various proteins, the principal substrate being retinoblastoma (RB) protein. When phosphorylated, RB releases the E2F transcription factor, which activates genes that are necessary for DNA synthesis and cell cycle progression from the G1 to S phase. In addition to its catalytic function, cyclin D1 also has noncatalytic functions, with the major function being transcriptional regulation 17. In prostate cancer, cyclin D1 has been shown to function as a corepressor to androgen receptor (AR) 18–20.

Because cyclin D1 is a suggested target of miR-193b in other cancers, we aimed to study whether it is also a target in prostate cancer. In addition, our goal was to confirm the hypermethylation of miR-193b in clinical prostate cancer samples.

## Materials and Methods

### Cell lines, xenografts, and clinical samples

The prostate cancer cell lines 22Rv1, PC-3, LNCaP, and DU145 were obtained from the American Type Cell Collection (Manassas, VA). LAPC-4 and VCaP cell lines were provided by Dr. Charles Sawyers (University of California at Los Angeles, Los Angeles, CA) and Dr. Jack Schalken (Radboud University Nijmegen Medical Center, Nijmegen, the Netherlands), respectively. All cell lines were cultured under recommended conditions. The 17 PC LuCaP xenografts were provided by one of the authors (R.L.V.).

All clinical samples were obtained from Tampere University Hospital (TAUH, Tampere, Finland). Fresh-frozen tissue samples, including 10 benign prostate hyperplasias (BPH), 26 untreated prostatectomy specimens, and nine castration-resistant tumors (CRPC), were used to study miR-193b methylation, whereas 78 hormonally untreated PC prostatectomy specimens (Table S1) were used to study miR-193b expression. BPH samples were obtained from transurethral resection (TURP) or (cysto)prostatectomies from patients with BPH or bladder cancer. Untreated cancer samples were obtained from prostatectomies and CRPC samples from TURP. The samples used in this study were histologically examined to contain >70% cancerous or hyperplastic tissue. For cyclin D1 immunohistochemical analysis, a total of 267 formalin-fixed prostate cancer specimens (198 from prostatectomies and 69 CRPC samples) were used to construct tissue microarrays (TMAs; Table S2). Ethics Committee of Tampere University Hospital and National Authority for Medicolegal Affairs have approved the use of clinical tumor material.

### DNA and RNA extraction from clinical samples

For the methylation analysis, freshly frozen tissue blocks were cut into 10 × 20-micrometer sections using a cryotome. DNA was isolated using an AllPrep RNA/DNA minikit (Qiagen, Valencia, CA) according to the manufacturer's protocol. For miR-193b expression analysis and arrays, RNA was isolated using Trizol® reagent (Invitrogen, Life Technologies Corporation, Carlsbad, CA) according to the manufacturer's protocol.

### Microarrays

miRNA and mRNA expression data from cell lines was produced using Agilent Tehcnologies’ (Santa Clara, CA) miRNA microarray system (version 1 array containing 470 human and 64 viral miRNAs) 8 and Whole Human Genome Kit (4 × 44k) (Agilent Tehcnologies, Santa Clara, CA), respectively. For LuCaP xenografts Affymetrix (Santa Clara, CA) HG U133 Plus 2.0 microarray was used. The arrays were done according to the manufacturer's protocols.

### Cell transfection and cotransfection

To determine the effect of miR-193b expression on cyclin D1, RB, and pRB levels, 22Rv1 cells were transfected with a final concentration of 5 nmol/L pre-miR-193b or scrambled control using INTERFERin transfection reagent (Polyplus-transfection, Illkirch, France) according to the manufacturer's instructions. The cells were collected 3 days after transfection. miRNA precursors were purchased from Ambion (Applied Biosystems/Ambion, Austin, TX).

For the Cyclin D1 rescue and cell cycle experiment, 22Rv1 cells were cotransfected with 10 *μ*g of plasmid, pCMV-CCND1 lacking a 3’UTR (Plasmid 19927; Addgene, Cambridge, MA), or the control plasmid pCMV6-AC-GFP (OriGene Technologies, Rockville, MD) along with either pre-miR-193b or pre-miR-scramble at a final concentration of 10 nmol/L, using jetPRIME transfection reagent (Polyplus-transfection). The cells were collected 2 days after transfection.

### Flow cytometric analysis

Cells were harvested by trypsinization, washed with phosphate-buffered saline (PBS) and fixed in cold 70% EtOH. Ethanol was aspirated, and the cells were washed and rehydrated with PBS. Staining was performed with propidium iodide (Sigma-Aldrich, St. Louis, MO), and cell cycle profiles were analyzed with Accuri C6 Flow cytometer.

### Luciferase reporter assay

For the luciferase reporter assay, the pSGG-3UTR plasmid was purchased from SwitchGear Genomics (Menlo Park, CA). The plasmid contains a luciferase gene fused with the 3’UTR of *CCND1*. 22Rv1 cells were cotransfected with the 3’UTR plasmid, a control plasmid-containing Renilla luciferase and pre-miR-193b or pre-miR-scramble using Lipofectamine™ 2000 transfection reagent (Invitrogen) according to the manufacturer's instructions. Firefly and Renilla luciferase activities were measured 24 h after transfection using the Dual-Glo Luciferase Assay System (Promega, Madison, WI). Renilla luciferase values were used for data normalization. The luciferase assay was performed in quadruplicate, and repeated four times.

### Western Blot

Nuclear and cytoplasmic proteins for RB and pRB western blots and total protein for cyclin D1 western blots were isolated as described previously 21,22. A total of 20 *μ*g of proteins were separated on 8% (RB, pRB) and 10% (Cyclin D1) SDS-PAGE gels and blotted to a PVDF membrane (Immobilon-P, Millipore Corp., Billerica, MA). The membranes were incubated with primary antibodies against phospho-Rb (Ser795, 1:1000, Cell Signaling, Danvers, MA), Rb (C-15: sc-50, 1:500; Santa Cruz Biotechnology, Inc., Dallas, TX) or cyclin D1 (clone SP4, 1:100; Dako, Glostrup, Denmark) and with antibodies against fibrillarin (C13C3, 1:4000; Cell Signaling) or actin (pan AB-5 clone ACTN05, 1:400; Lab Vision Corp., Fremont, CA). After secondary antibody incubation (anti-rabbit-HRP for phospho-RB, RB, cyclin D1 and fibrillarin, and anti-mouse-HRP for Actin; 1:3000, Dako), the proteins were visualized by autoradiography.

### Immunohistochemistry

Antibodies against cyclin D1 (dilution 1:100, clone SP4; Dako) and Ki-67 (dilution 1:500, MM1; Leica Biosystems Newcastle Ltd., Newcastle upon Tyne, UK) were used with a Power Vision+ Poly-HRP IHC kit (ImmunoVision Technologies Co., Hillsborough, CA) according to the manufacturer's instructions and as described by Leinonen et al. 23. The slides were scanned with an Aperio ScanScope XT scanner (Leica Microsystems GmbH, Wetzlar, Germany). The virtual microscope 24,25 and the ImmunoRatio web application 26 were used to score the cyclin D1-staining intensity (0–1 = negative and weak, 2 = moderate, and 3 = strong) in a blinded fashion.

### Quantitative real-time RT-PCR

For miR-193b q-RT-PCR, a TaqMan microRNA Assay (Applied Biosystems, Foster City, CA) and the CFX96 q-RT-PCR detection system (Bio-Rad Laboratories Inc., Hercules, CA) were used according to the manufacturers’ recommendations. miRNA expression was normalized to RNU6B expression. For *CCND1* q-RT-PCR, first-strand complementary DNA synthesis was performed from total RNA using AMV reverse transcriptase (Finnzymes Inc., Espoo, Finland) according to the manufacturer's instructions. The expression of *CCND1* was measured with Maxima SYBR Green (Fermentas Inc., Burlington, ON, Canada) and the CFX96 q-RT-PCR detection system (Bio-Rad Laboratories Inc.) and was normalized to the *β*-Actin reference gene. The primer sequences used for the *CCND1* and *β*-Actin q-RT-PCR were: CyclinD1for 5’-CCCTCGGTG GGTCCTACTTCAA-3’, CyclinD1rev 5’-TGGCATTTTGG AGAGGAAGT-3’, and Bactinf4 5’-TGGGACGACATGGAG AAAAT-3’, Bactinr4 5’-AGAGGCGTACAGGGATAGCA-3’.

### miR-193b methylation analysis

Methylated DNA was enriched using MethylMiner™ Methylated DNA Enrichment Kit (Invitrogen) according to the manufacturer's instructions. Briefly, 2 *μ*g of genomic DNA was fragmented by sonication. Methylated DNA was enriched by binding to magnetic beads coated with the methyl-CpG-binding domain of the human MBD2 protein (Methyl-CpG Binding Domain Protein 2) and eluted as a single fraction using 2 M NaCl. Finally, the DNA was ethanol-precipitated and resuspended in 50 *μ*L of DNase-free water. For q-PCR, iQTM SYBR® Green supermix and CFX96 q-RT-PCR detection system (Bio-Rad Laboratories Inc.) were used. The sequences of the primers used in the q-PCR were: miR-193b_DMR_f1 5’-TGGCGTTTCTGG TTTCTCTT-3’ and miR-193b_DMR_r2 5’-CGCACCTTTTCTCCTCATTT-3’. Each sample was run in duplicate. The methylation status of miR-193b was calculated from the elution-fraction signal in relation to the total q-PCR signal.

### Growth curves

For growth analysis with the CDK4/6 inhibitor PD0332991 (Selleck Chemicals, Houston, TX), the 22Rv1 cells were seeded in 24-well plates in quadruplicate for each inhibitor concentration. The following day, the cells were imaged (day 0), and then treated with 0 nmol/L, 100 nmol/L, 500 nmol/L, or 2000 nmol/L inhibitor. The medium was replaced every other day with fresh medium containing the inhibitor. The cells were imaged daily with an Olympus IX71 microscope with the OASIS automation control system and Surveyor imaging software version 5.5.5.26 (Objective Imaging Ltd., Cambridge, UK). Cell growth was analyzed by measuring the cell surface area with an in-house macro and ImageJ software (NIH, Bethesda, MD). The experiments were repeated three times.

### Statistical analysis

An unpaired *t* test was used to calculate the significant difference in cell cycle analysis and in luciferase activity between the pre-miR-scramble and pre-miR-193b transfected samples as well as the difference in miR-193b expression between differentially staged prostatectomy tumors. The paired *t* test was used to calculate the *P*-value of the growth curve assays on the last day of the experiment. Fisher's exact test was used to assess the significant difference in miR-193b methylation in clinical samples. Fisher's exact, chi-square, Mann–Whitney U, and unpaired *t* tests were used to analyze the association between clinicopathologic variables. A *P*-value <0.05 was considered significant. Spearman's correlation was used to study the correlation of miR-193b and *CCND1* expression in cell lines and xenograft samples in microarray analysis.

## Results

**Figure 1:**
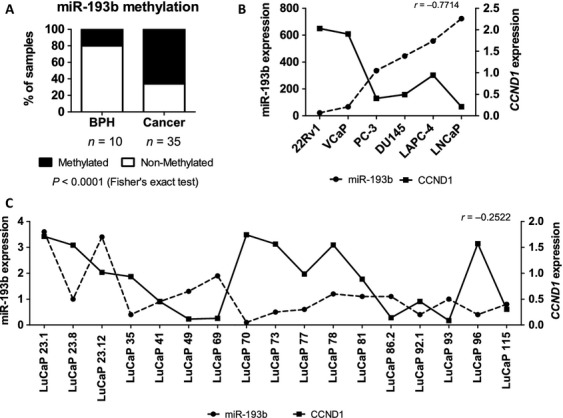
miR-193b is methylated in cancer samples and has inverse expression pattern compared to *CCND1*. (A) miR-193b methylation in clinical samples (BPH, PC, and CRPC) was assessed by MethylMiner-qPCR. miR-193b and *CCND1* expressions were studied by miRNA and mRNA microarray (B) in prostate cancer cell lines and (C) xenograft samples. Spearman correlation coefficiencies are given. BPH, benign prostate hyperplasias; CRPC, castration-resistant tumors; PC, prostate cancer.

We have previously shown that miR-193b is strongly hypermethylated in 22Rv1 cells and moderately methylated in VCaP cells but is not methylated in LAPC-4, LNCaP, DU145, PC-3, EP156T, or PrEC cells, as determined by bisulfite sequencing 8. In addition, we sequenced five untreated and four CRPC clinical specimens and found increased methylation 8. Here, we wanted to confirm hypermethylation of miR-193b in clinical specimens. We recently performed genome-wide methylation analysis of methylation by MeDIP-sequencing (unpubl. data) and found that the most differentially methylated genomic region in proximity to miR-193b is 16:14396975–14397475 (GRCh37), which is located 349 bp upstream of the miR-193b gene. Here, we measured the methylation of miR-193b in that region in BPH (*n* = 10), PC (*n* = 26) and CRPC (*n* = 9) samples using MethylMiner-qPCR. The samples were classified as methylated if they showed more than 20% methylation. According to our analysis, miR-193b was significantly more methylated in cancer than in BPH (*P *<* *0.0001; Fig.1A).

Next, we measured the expression of miR-193b in a larger set (*n* = 78) of prostatectomy samples and found that the expression was lower (*P *<* *0.05) in stage pT3 tumors than in stage pT2 tumors (Fig. S1). However, we did not find an association between miR-193b expression and the Gleason score or progression-free survival (data not shown).

**Figure 2:**
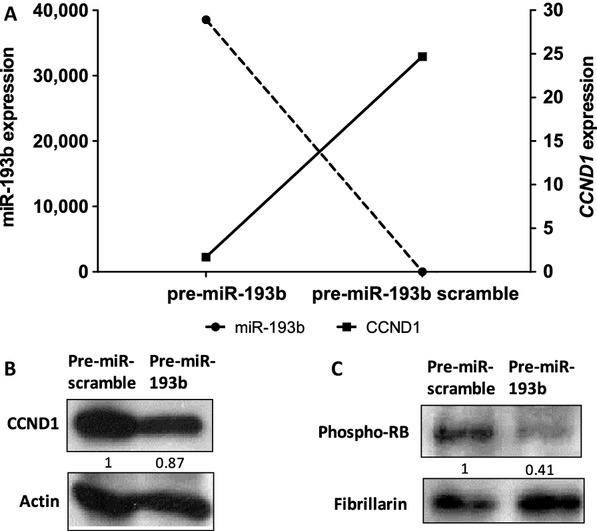
miR-193b overexpression reduces the expression of *CCND1*, the level of Cyclin D1 protein and phosphorylation level of retinoblastoma (RB). 22Rv1 prostate cancer cells were transfected with pre-miR-193b or pre-miR-control. (A) Expression levels of *CCND1*mRNA was measured using q-RT-PCR. Western blot analysis was used to detect protein expression of (B) Cyclin D1 from total proteins and (C) phosphorylation level of RB protein from nuclear protein fraction. Actin and fibrillarin antibodies were used as loading controls.

Because *CCND1* is a suggested target of miR-193b target in hepatocellular and pancreatic carcinoma and melanoma 10,11,14, we aimed to determine if it is also a target in prostate cancer. The expression levels of miR-193b and *CCND1* were obtained using expression microarrays of PC cell lines and xenograft samples. The array data showed a negative correlation between miR-193b and *CCND1* expression in the cell lines (Fig.1B, *r* = −0.7714) as well as in the xenograft samples (Fig.1C, *r* = −0.2522). We then studied the expression of *CCND1* mRNA and protein levels in 22Rv1 cells transiently transfected with pre-miR-193b. A marked reduction in the *CCND1* expression at both the mRNA (Fig.2A) and protein (Fig.2B) level was detected in cells transfected with pre-miR-193b compared to the scrambled pre-miR transfected cells. Similar to 22Rv1 cells, the Cyclin D1 protein expression was diminished also in VCaP cells transiently transfected with pre-miR-193b (Fig. S2). In addition, a marked reduction was detected in phospho-RB protein level in nuclear protein fractions (Fig.2C).

**Figure 3:**
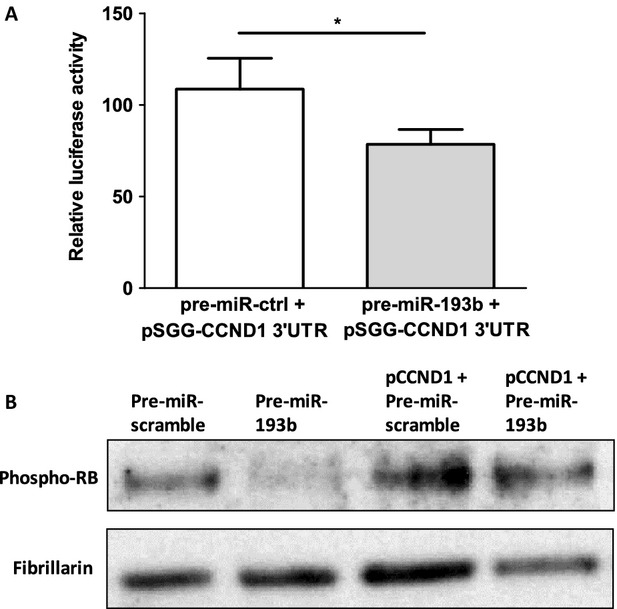
miR-193b targets *CCND1* 3’UTR. (A) Luciferase experiment was performed in 22Rv1 cells cotransfected with pSGG-plasmid containing *CCND1* 3’UTR, Renilla luciferase plasmid, and pre-miR-193b or pre-miR-control. Values were normalized against Renilla luciferase activity. The means of four experiments ±SEM are shown. *P-value <0.05 (B) Western blot analysis of pRB in 22Rv1 cells transfected with pCMV-CCND1 plasmid lacking *CCND1* 3’UTR together with miR-scramble or miR-193b. Fibrillarin antibody was used as loading control for nuclear proteins.

To confirm that miR-193b binds to the 3’UTR -region of the *CCND1* gene in prostate cancer cells, we performed a luciferase reporter assay. A pSGG-luciferase plasmid containing *CCND1* 3’UTR at the 3’ end of the luciferase gene was transfected together with either pre-miR-193b or pre-miR-scramble into 22Rv1 cells which express low levels of miR-193b endogenously. The inhibition of luciferase activity was observed when the cells were transfected together with the plasmid and pre-miR-193b (*P *<* *0.05) but not with pre-miR-scramble (Fig.3A), confirming that miR-193b targets the 3’UTR of *CCND1*.

To evaluate the significance of *CCND1* as a miR-193b target, a rescue experiment was performed. We used the phosphorylation of RB as a measure of cyclin D1 activity. Phospho-RB levels decreased in cells transfected with pre-miR-193b. When the cells were cotransfected with pre-miR-193b and the pCMV-CCND1 plasmid lacking the 3’UTR of *CCND1*, no such reduction in phospho-RB levels was observed (Fig.3B). Similarly, transient pre-miR-193b transfection caused a reduction in the number of cells in S and G2/M phase fractions of cell cycle, whereas there was no difference in cells cotransfected with pre-miR-193b and *CCND1* lacking 3’UTR (Fig. S3).

**Figure 4:**
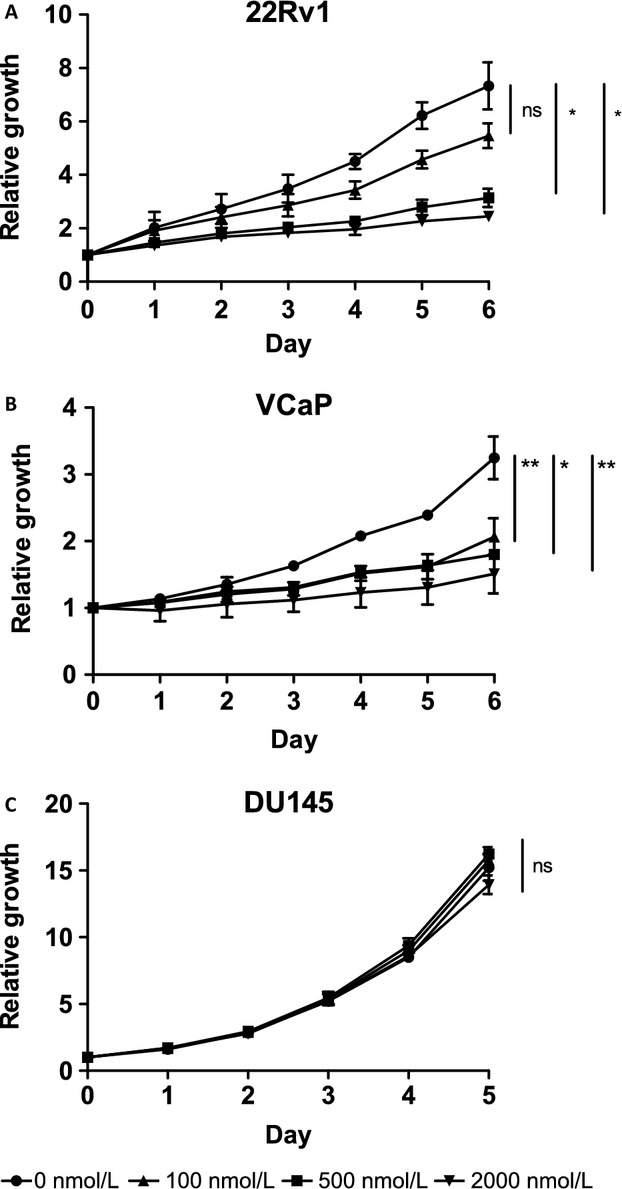
CDK 4/6 inhibitor PD0332991 suppresses the growth of (A) 22Rv1 and (B) VCaP cells but not (C) DU145 cells. Cells were treated with 0, 100, 500, and 2000 nmol/L concentrations of the inhibitor and growth was followed for 5 to 6 days. Each concentration was done in quadruplicates and each experiment was done in triplicates, averages from experiments ±SEM are shown. *P*-values of growth differences between different concentrations on day 5 or 6 were calculated using paired *t*-test, *P-value <0.05, **P-value <0.01.

Because cyclin D1 regulates the activity of CDK4/6, we treated PC cell lines with the CDK4/6 inhibitor PD0332991 at different concentrations and measured the effect on prostate cancer cell growth. 22Rv1 and VCaP cells, which express low levels of miR-193b and high levels of *CCND1*, showed significant growth suppression at a 500 nmol/L concentration (*P *<* *0.05; Fig.4A, B), whereas the various concentrations of the inhibitor had little effect on the growth of DU145 (Fig.4C), LNCaP LAPC-4 or PC-3 cells, which express high levels of miR-193b and low levels of *CCND1* (Fig. S4A–C). Figure S4D shows that the vehicle, dimethylsulfoxide (DMSO), has no effect on the cell growth of 22Rv1 cells at 0.4% concentration.

To study the expression of cyclin D1 in clinical samples, we performed an immunohistochemical analysis on TMAs. The analysis showed that the CRPC samples expressed higher levels of cyclin D1 compared to PC samples (*P *=* *0.0237; Table1). In prostatectomy samples, the cyclin D1 staining intensity was not associated with Gleason score, pT stage, or diagnostic PSA levels. However, there was strong positive association between cyclin D1 and the proliferation marker Ki-67 (*P *<* *0.0001) in the prostatectomy cohort. CRPC cells expressing high levels of cyclin D1 had increased Ki-67 values, although the association was not statistically significant.

**Table 1:** Association of clinicopathological variables with Cyclin D1 expression

Variable	Cyclin D1 expression		*P*
	Negative (0/1)	Positive (2–3)	
Prostatectomy specimens, *n* (%)	74 (37)	124 (63)	
Locally recurrent CRPCs, *n* (%)^1^	20 (29)	49 (71)	0.0237
Prostatectomy specimens			
Gleason score, *n* (%)^1^			
<7	24 (33)	46 (37)	
7	38 (52)	60 (48)	
>7	11 (15)	19 (15)	0.8337
pT Stage, *n* (%)^2^			
pT2	54 (75)	88 (72)	
pT3	18 (25)	35 (28)	0.6216
PSA ng/mL (mean ± SD)^3^	20.0 ± 31.5	14.3 ± 11.3	0.9786
Age (mean ± SD)^4^	62.6 ± 5.2	63.2 ± 4.9	0.4167
Ki-67 (mean ± SD)	7.1 ± 7.0	13.6 ± 14.5	<0.0001
Locally recurrent CRPCs, *n* (%)^3^			
Ki-67 (mean ± SD)	13.2 ± 9.3	20.7 ± 15.8	0.0950

1 Chi-square test.

2 Fisher's exact test.

3 Mann–Whitney *U*-test.

4 Unpaired *t* test.

## Discussion

Here, we confirmed the hypermethylation of miR-193b gene in prostate cancer. The hypermethylation of miR-193b leads to reduced expression, as we have previously shown 8. Because miRNAs regulate the expression of protein-coding genes, the key question is which proteins are targeted by miR-193b in prostate cancer. *CCND1* is a target in hepatocellular and pancreatic carcinoma, as well as in melanoma 10,11,14. We have previously shown that the overexpression of miR-193b reduces the proliferation of prostate cancer cells due to a decreased number of cells in S-phase of the cell cycle 8, suggesting that *CCND1* could also be a target for miR-193b in prostate cancer.

We showed that the expressions of miR-193b and *CCND1* are inversely correlated in prostate cancer cell lines and xenografts. Subsequently, we demonstrated reduced mRNA and protein expression of *CCND1* in 22Rv1 cells transiently transfected with pre-miR-193b. Using a reporter assay, we confirmed that miR-193b targets the 3’UTR of the *CCND1* gene in 22Rv1 prostate cancer cells. Concordantly, Chen et al. 11. and Xu et al. 10. have previously shown by luciferase assay that miR-193b targets the *CCND1* 3’UTR in Malme-3M malignant melanoma and HepG2 hepatocellular carcinoma cells. In addition, we performed a rescue experiment, in which we cotransfected miR-193b into 22Rv1 cells with and without the pCMV-CCND1 plasmid lacking the 3’UTR of *CCND1*. These results show that *CCND1* is a bona fide target of miR-193b in prostate cancer cells.

To assess the relevance of cyclin D1 targeting by miR-193b in prostate cancer cells, we studied the activity of the cyclinD1–RB pathway in the regulation of the G1/S transition in the cell cycle. The transfection of 22Rv1 cells with miR-193b reduced the level of phospho-RB, in accordance with the known function of cyclin D1 in the regulation of RB phosphorylation. In addition, the phosphorylated RB protein levels were rescued in pCMV-CCND1/miR-193b cells to those of the control cells. Finally, when prostate cancer cell lines were treated with the CDK4/6 inhibitor PD0332991, the cell lines responded to the treatment according to miR-193b expression/methylation status and cyclin D1 levels. Those with low miR-193b and high cyclin D1 (22Rv1, VCaP) responded to the drug with growth inhibition, while the others did not. These results demonstrate that the downregulation of cyclin D1 by miR-193b is functionally relevant for prostate cancer cell growth.

To assess the clinical significance of cyclin D1, we stained TMAs and found that the expression of cyclin D1 is higher in CRPC (*n* = 69) compared to hormone-naïve PC (*n* = 198). Previously, Drobnjak et al. 27. showed increased cyclin D1 staining in 22 CRPC bone metastases compared to 86 primary PC tumors. In our prostatectomy samples, the expression was strongly associated with proliferation but not with Gleason score, pathological status, PSA value, or age at diagnosis. This is in line with previous studies showing that cyclin D1 expression is associated with the proliferation marker Ki-67 but not with other clinicopathological variables 27–31.

Because it is known that one gene can be targeted by several miRNAs and that cyclin D1 is a key regulator of the cell cycle G1/S transition, it is not surprising that *CCND1* has been suggested to be a target of more than one miRNA in prostate cancer. Bonci et al. reported that miR15-a and 16-1 interact directly with the 3’UTR of *CCND1* and reduce cyclin D1 protein expression, thereby reducing prostate cell proliferation 32. However, the function of *CCND1* targeting by these three miRNAs is altered by different mechanisms in prostate cancer. miR-193b is epigenetically regulated and silenced by methylation 8, whereas the function of miRs 15-a and 16-1 is disrupted by deletions in the encoding chromosomal region 13q14 32. We have previously reported a homozygous deletion of the miR15-a and 16-1 locus in prostate cancer, although the frequency is relatively low 33.

In addition to *CCND1*, there are several other suggested targets for miR-193b, such as *YWHAZ*,*PLAU*(aka *uPA*), and *KIT*in different cancer types 9–16. Xie et al. 34. showed that the knockdown of cystic fibrosis transmembrane conductance regulator (CFTR) led to the suppression of miR-193b expression. They also showed that the forced overexpression of miR-193b completely abrogated elevated urokinase-type plasminogen activator (uPA) activity after CFTR-knockdown in PC-3 cells, suggesting that the tumor-suppressing effect of CFTR is mediated through the miR-193b-uPA axis. However, it should be noted that miR-193b expression is low in 22Rv1 and VCaP cells 8, which do not express uPA 35,36, suggesting that uPA is not the major target of miR-193b in prostate cancer.

In conclusions, we have demonstrated that the overexpression of cyclin D1 in prostate cancer is driven, at least partly, by the reduced expression of miR-193b. The mechanism for miR-193b suppression is the hypermethylation of the DMR upstream of the miR-193b gene. Additional studies are warranted to translate these findings to clinical benefits. For example, the restoration of miR-193b expression could theoretically reduce the proliferation of prostate cancer cells. Alternatively, the loss of miR-193b expression could indicate the sensitivity of prostate cancer cells to cyclin D1 inhibition.

## Supporting Information

Table S1. Description of clinical samples used to study miR-193b expression. **Table S2.** Description of clinical samples used in immunohistochemistry. **Figure S1.** miR-193b expression in clinical samples. **Figure S2.** miR-193b overexpression reduces the expression of *CCND1*in protein level in VCaP cells. **Figure S3.***CCND1*lacking 3’ UTR is able to rescue cell cycle effects of miR-193b. **Figure S4.** The effect of CDK 4/6 inhibitor PD0332991 and dissolvent dimethylsulfoxide (DMSO) on growth of (A) LAPC-4, (B) LNCaP, (C) PC-3, and (D) 22Rv1 cells.
