# Supplementary material for: Epigenetically altered miR-193b targets cyclin D1 in prostate cancer
Source: Cancer Med. 2015 Jul 1;4(9):1417–25. doi: 10.1002/cam4.486 (PMC4567026; doi:10.1002/cam4.486)
Supplement: Table S1. — Description of clinical samples used to study miR-193b expression. Table S2. Description of clinical samples used in immunohistochemistry. Figure S1. miR-193b expression in clinical samples. Figure S2. miR-193b overexpression reduces the expression of CCND1in protein level in VCaP cells. Figure S3.CCND1lacking 3’ UTR is able to rescue cell cycle effects of miR-193b. Figure S4. The effect of CDK 4/6 inhibitor PD0332991 and dissolvent dimethylsulfoxide (DMSO) on growth of (A) LAPC-4, (B) LNCaP, (C) PC-3, and (D) 22Rv1 cells. [file cam40004-1417-sd1.docx]

**Supplementary material to**

**Epigenetically Altered miR-193b Targets Cyclin D1 in Prostate Cancer**

Kirsi M. Kaukoniemi^1*,2^, Hanna E. Rauhala^1*^, Mauro Scaravilli^1,2^, Leena Latonen^1,2^, Matti Annala^1^, Robert L. Vessella^3^, Matti Nykter^1^, Teuvo L.J. Tammela^4^, and Tapio Visakorpi^1,2^.

**Supplementary table 1.** Description of clinical samples used to study miR-193b expression.

| Prostatectomy specimens, n | 78 |
| --- | --- |
| Gleason score, n (%) |  |
| <7 | 33 (42) |
| 7 | 32 (41) |
| >7 | 13 (17) |
| pT stage, n (%) |  |
| pT2 | 46 (59) |
| pT3 | 32 (41) |
| Mean PSA ng/mL | 11.8 (range: 3.15-51.5) |
| Mean age at diagnosis | 62.1 years (range: 47.4-71.8) |

**Supplementary table 2. Description of clinical samples used in immunohistochemistry.**

| Prostatectomy specimens, n | 198 |
| --- | --- |
| Gleason score, n (%) |  |
| <7 | 70 (35) |
| 7 | 98 (50) |
| >7 | 30 (15) |
| pT stage, n (%) |  |
| pT2 | 142 (72) |
| pT3 | 53 (27) |
| Mean age at diagnosis | 63 years (median 63, range 44-74 yrs) |
| Median follow-up time | 76 months (range 4.9-218.7 mo) |
|  |  |
| Locally recurrent CRPCs, n | 69 |
| Mean time from the beginning of treatment to the TURP | 43 months (range 3-144 mo) |

**Supplementary figure S1.**


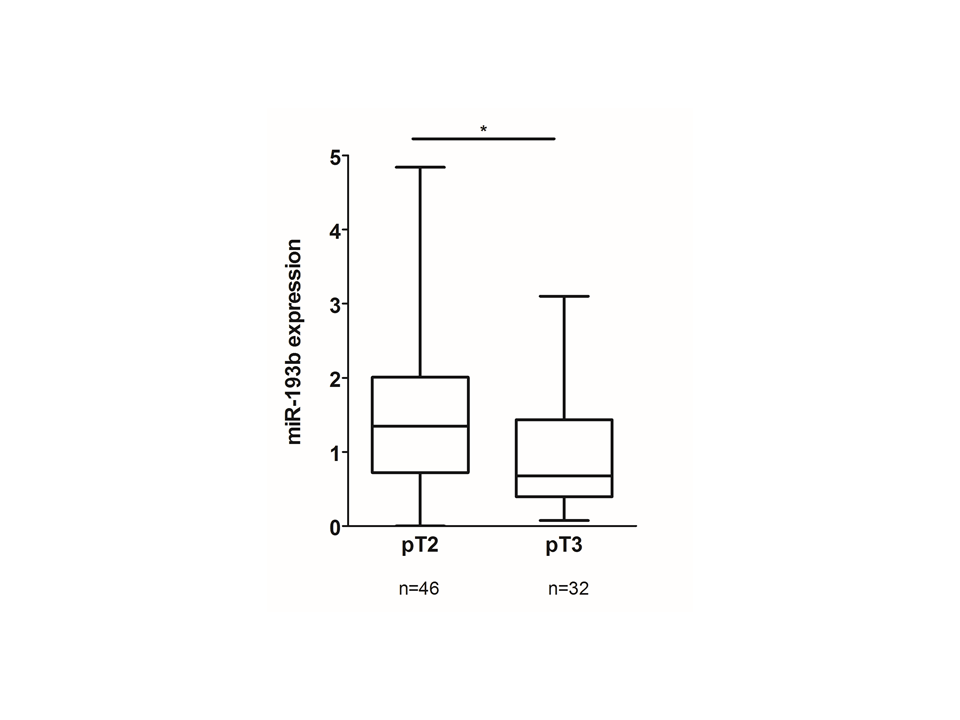


**Figure S1. miR-193b expression in clinical samples.** miR-193b expression was analyzed from pathological stage 2 and 3 prostatectomy samples using q-RT-PCR. P-value was calculated with t-test, * *p*-value <0.05.

**Supplementary figure S2.**


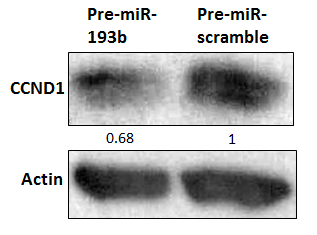


**Figure S2. miR-193b overexpression reduces the expression of *CCND1* in protein level in VCaP cells.** VCaP cells were transiently transfected with pre-miR-193b or pre-miR-ctrl and Western blot analysis was used to detect the protein expression of Cyclin D1 from total proteins.

**Supplementary figure S3.**

**
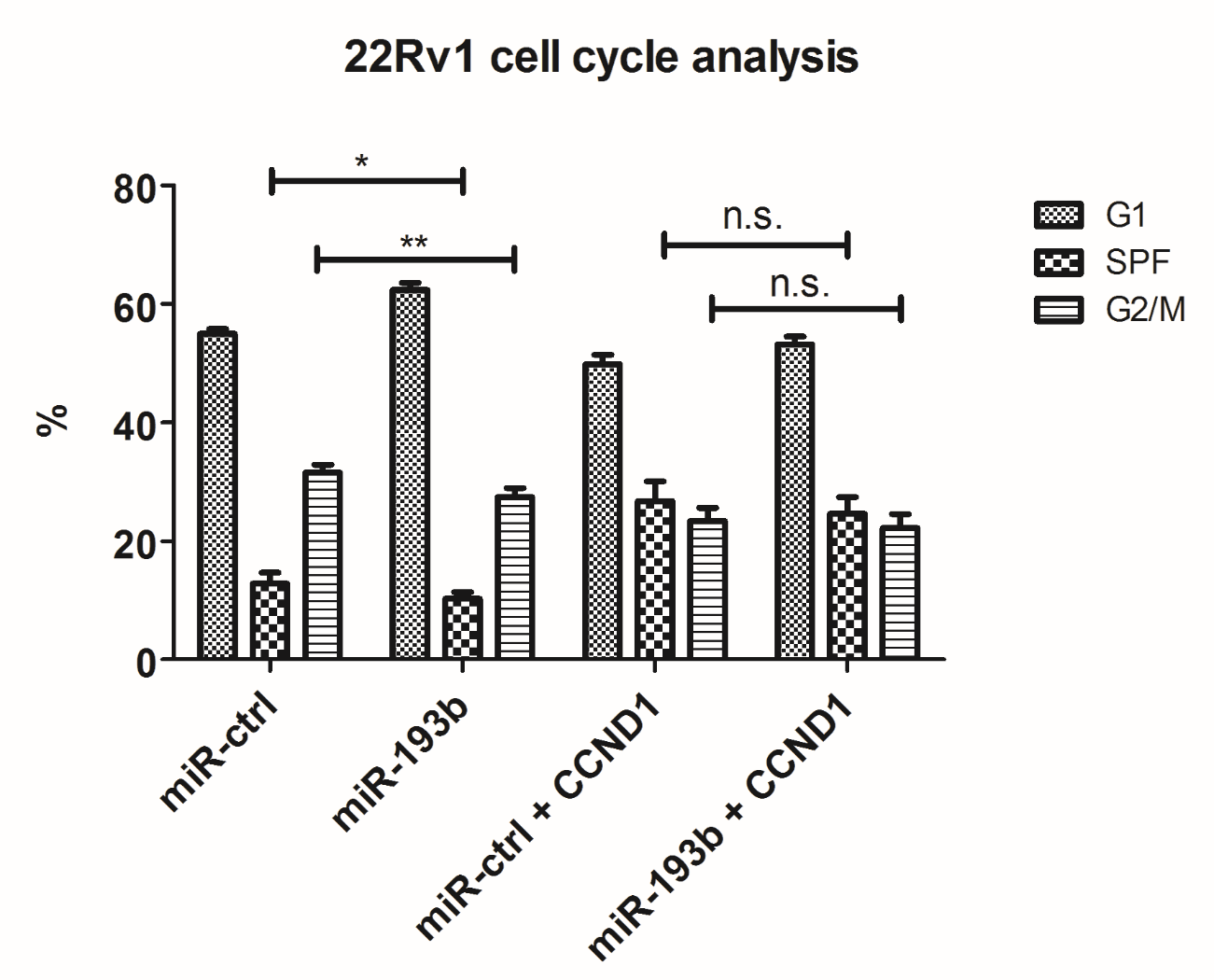
**

**Figure S3. *CCND1* lacking 3’ UTR is able to rescue cell cycle effects of miR-193b.** miR-193b decreases the proportion of cells in S and G/M2 phases of cell cycle (*SPF *p*-value <0.032, **G2/M *p*-value 0.028), whereas expression of *CCND1* without 3’UTR (pSGG-CCND1 3’UTR) together with miR-193b abolishes miR-193b effect in cell cycle phases S and G2/M.

**Supplementary figure S4.**


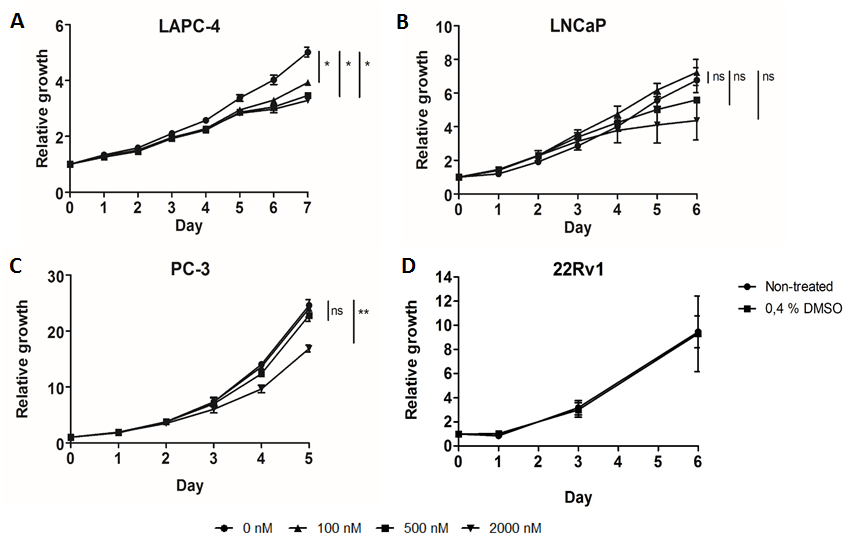


**Figure S4.** **The effect of** **CDK 4/6 inhibitor PD0332991 and dissolvent dimethylsulphoxide (DMSO) on growth of (A) LAPC-4, (B) LNCaP, (C) PC-3 and (D) 22Rv1 cells.** LAPC-4 (A), LNCaP *(*B), and PC-3 (C) cells were treated with 0, 100, 500 and 2000 nM concentrations of the inhibitor. 22Rv1 cells were treated 0.4% of DMSO to find out whether the dissolvent alone has an effect on cell growth. Cells were imaged before starting the treatment and growth was followed for 5 to 6 days. Each concentration was done in quadruplicates and each experiment was done in triplicates, averages from experiments ± SEM are shown. P-values from growth differences between different concentrations on day 5 or 6 were calculated using paired t-test, * *p*-value <0.05, ** *p*-value <0.01.
